# Tumor necrosis factor α inhibitor-induced alopecia in pediatric patients: a cohort of 20 patients and review of the literature

**DOI:** 10.1007/s00403-025-04300-0

**Published:** 2025-06-06

**Authors:** Shiran Reiss-Huss, Daniel Hilewitz, Sharon Yacobovitz, Manar Matar, Yael Weintraub, Dror S. Shouval, Chani Topf-Olivestone, Lev Pavlovsky, Rotem Tal, Gil Amarilyo, Yael Renert-Yuval, Rivka Friedland

**Affiliations:** 1 Pediatric Dermatology Unit, Schneider Children’s Medical Center of Israel, Petah Tikva, Israel; 2 School of Medicine, Faculty of Medical and Health Sciences, Tel Aviv University, Tel Aviv, Israel; 3 Institute of Gastroenterology, Nutrition and Liver Diseases, Schneider Children’s Medical Centre, Petach-Tikva, Israel; 4 Pediatric Gastroenterology Clinic, Assuta Medical Center Ashdod, Ashdod, Israel; 5 Division of Dermatology, Rabin Medical Center, Petah Tikva, Israel; 6 Pediatric Rheumatology Unit, Schneider Children’s Medical Centre, Petach-Tikva, Israel

**Keywords:** Child, Alopecia, Drug-related side effects and adverse reactions, Tumor necrosis factor inhibitors

## Abstract

Anti TNFα agents can induce cutaneous adverse events in both adults and children. While drug-related alopecia was reported in adults treated with TNFα inhibitors for various indications, pediatric data are scarce. To describe clinical characteristics and outcomes in pediatric patients with TNFα inhibitor-induced alopecia we conducted a single center retrospective study (0748-21-RMC, retrospectively registered on January 2nd 2022) including all patients aged < 18 years who were treated with TNFα inhibitors for any indication and developed drug-induced alopecia between the years 2018–2023. A comprehensive literature review was also performed. Twenty patients were included (mean age 12.9 ± 3.1 years, male:female ratio 1:1.4). Fourteen were diagnosed with Crohn’s disease, three with ulcerative colitis, and three with juvenile idiopathic arthritis. Half of the patients were treated with adalimumab and half with infliximab. Overall, alopecia was observed after 14.8 ± 10.8 months of treatment. Eighteen (90.0%) patients presented with psoriatic-like inflammatory alopecia, and two with alopecia areata-like lesions. Seventeen (85.0%) patients discontinued their anti-TNFα therapy due to the alopecia, all presented hair regrowth within six months. Hair regrowth was not recorded in three patients who continued TNFα inhibitors. Literature review of pediatric TNFα inhibitor-induced alopecia revealed comparable patients’ demographics and response to treatment discontinuation. In conclusion, TNFα inhibitor-induced alopecia is a rare adverse event in children, occurring mainly in adolescents with inflammatory bowel diseases. Our relatively large cohort provides further evidence for the need for TNFα inhibitor cessation to improve drug-induced alopecia in pediatric patients.

## Introduction

TNFα inhibitors (TNFαI) are indicated for several inflammatory diseases in children, including psoriasis, hidradenitis suppurativa, uveitis, inflammatory bowel disease (IBD) and juvenile idiopathic arthritis (JIA) [15, 16]. The use of these monoclonal antibodies has changed the treatment paradigm for severe debilitating diseases in the pediatric population, but also revealed new cutaneous adverse events [28]. TNFαI-induced alopecia variants in adults include inflammatory alopecia (IA), i.e., clinical manifestation mimicking psoriatic alopecia with erythema and scales, alopecia areata (AA)-like presentation [1, 7, 10, 21, 28, 30], lichen planopilaris-like alopecia [17, 19] and nonspecific inflammatory alopecia [20]. These cases can be severe, refractory and significantly impact patients’ quality-of-life. Notably, there are limited data regarding TNFαI-induced alopecia in the pediatric population, with several case reports and small case series reported in the literature [4, 5, 8, 14, 23, 25, 26].

Herein we describe the clinical characteristics and outcomes of a relatively large cohort of 20 pediatric patients from a single tertiary pediatric medical center presenting with TNFαI-induced alopecia, and compare our cohort’s results with a comprehensive literature review.

## Methods

This is a retrospective cohort study. Inclusion criteria were patients aged ≤ 18 years who were diagnosed clinically by a pediatric dermatologist with IA or AA while treated with a TNFαI and followed for at least 12 months after diagnosis. The diagnosis was made by a pediatric dermatologist based on clinical and dermoscopic findings. For IA, clinical findings included erythematous plaques and alopecia, with or without scales, and the dermoscopic findings included atrichia with or without scales, doted vessels and red globules. For AA-like eruption, clinical findings included alopetic plaques with no erythema or scales, with or without madarosis, with tapering hairs and yellow dots dermoscopically. Visits took place at the pediatric dermatology unit of a tertiary pediatric medical center between 2018 and 2023.

The study was conducted in accordance with the Declaration of Helsinki and approved by the local Institutional Review Board (0748-21-RMC) and registered with the Israel Ministry of Health.

Medical charts were reviewed for the following data: age, gender, personal and family history, medications, therapeutic management, and outcomes.

Complete resolution was defined as ≥ 90% hair regrowth, partial resolution as 50–89% hair regrowth, and no response was defined as < 50% hair regrowth [18]. Regrowth was assessed by clinical estimation and photography.

Continuous variables are presented as means and standard deviation (SD). Categorical variables are presented with adjusted frequencies.

A narrative review of the literature was conducted for all studies reporting therapeutic management for TNFαI-induced alopecia in pediatric patients. We searched for English language publications using PubMed. The following search terms was used: “TNF alpha inhibitors”, “adalimumab”, “infliximab”, “pediatric inflammatory bowel disease”, “pediatric ulcerative colitis”, “pediatric Crohn’s disease”, “psoriasis”, “psoriasiform dermatitis” and “alopecia”. We included all original studies of any design reporting patients younger than 18 years diagnosed with TNFαI-induced alopecia and the therapeutic management.

## Results

Twenty patients were included in this cohort. Demographic and clinical characteristics are presented in Table 1.

**Table 1:** Demographic and clinical characteristics, management and outcome

Pt No	Sex/age at alopecia onset (years)	Baseline medical condition	TNFα inhibitor	Clinical presentation: scalp/skin	Tx duration until alopecia onset (months)	Management	Outcome of alopecia	IBD/JIA activity at alopecia presentation	IBD/JIA Activity after Tx replacement	2nd line Tx
1	M/16	CD	Adalimumab	IA/pso derm	12	Ustekinumab	CR	Remission	Active	Upadacitinib
2	M/14	CD	Adalimumab	IA/pso derm	25	Ustekinumab	CR	Remission	Active	Risankizumab
3	M/15	CD	Infliximab	IA/pso derm	2	Continued infliximab, MTX, acitretin	NR	Active	N/A	
4	F/14	CD	Adalimumab	IA/pso + foll derm	12	Ustekinumab	CR	Remission	Active	Increased dosage
5	M/14	CD	Adalimumab	IA/Pso + Foll	48	Risankizumab	CR	Remission	Remission	
6	M/13	CD	Infliximab	IA/foll derm	17	Risankizumab	CR	Remission	Remission	
7	F/12	CD	Infliximab	IA/pso derm	12	Ustekinumab	CR	Remission	Remission	
8	F/12	CD	Infliximab	IA/pso + foll derm	13	Ustekinumab	CR	Remission	Remission	
9	F/16	CD	Adalimumab	AA-like/none	12	Risankizumab	CR	Active	Remission	
10	F/16	CD	Adalimumab	AA-like/none	24	Risankizumab	CR	Active	Remission	
11	M/11	CD	Adalimumab	IA/pso derm	24	Continued adalimumab, Topical Tx, MTX	NR	Remission	N/A	
12	M/16	CD	Adalimumab	IA/pso derm	24	Upadacitinib	CR	Remission	Remission	
13	F/12	CD	Infliximab	IA/foll derm	4	Ustekinumab	PR	Remission	Remission	
14	F/11	CD	Infliximab	IA/pso + foll derm	12	Continued adalimumab, MTX	NR	Remission	N/A	
15	F/12	UC	Infliximab	IA/pso derm	24	Vedolizumab	CR	Active	Remission	
16	F/13	UC	Infliximab	IA/pso derm	11	Tofacitinib	CR	Active	Remission	
17	F/12	UC	Infliximab	IA/pso derm	5	Vedolizumab	CR	Active	Remission	
18	M/18	JIA	Adalimumab	IA/foll derm	2	Secukinumab	CR	Remission	Active	Upadacitinib
19	F/6	JIA	Infliximab	IA/pso + foll derm	10	Tofacitinib	CR	Active	Remission	
20	F/6	JIA	Adalimumab	IA/pso derm	2	Tofacitinib	CR	Remission	Remission	

*AA* alopecia areata, *Abx* antibiotics, *CD* Crohn’s disease, *CR* complete resolution, *F* female, *Foll* follicular, *IA* inflammatory alopecia, *IBD* inflammatory bowel disease, *JIA* juvenile idiopathic arthritis, *M* male, *MTX* methotrexate, *N/A* not available, *NR* no resolution, *PR* partial resolution, *Pso* derm psoriasiform dermatitis, *Pt*. *No*. patient number, *Tx* treatment, *UC* ulcerative colitis

Seventeen patients (85.0%) were diagnosed with IBD: 14 with Crohn’s disease (CD) and three with ulcerative colitis (UC); and three patients had JIA.

Mean age at alopecia diagnosis was 12.9 ± 3.1 years (range 6–18 years) and male to female ratio was 1:1.4.

None of the patients had a prior personal history of psoriasis or AA, while a family history of psoriasis was reported in two patients.

Ten patients (50.0%) were treated with adalimumab and 10 (50.0%) with infliximab, none of them had taken concurrent medications.

Alopecia was diagnosed after a mean duration of 14.8 ± 10.8 months of treatment (range 2–48 months).

**Fig. 1:**
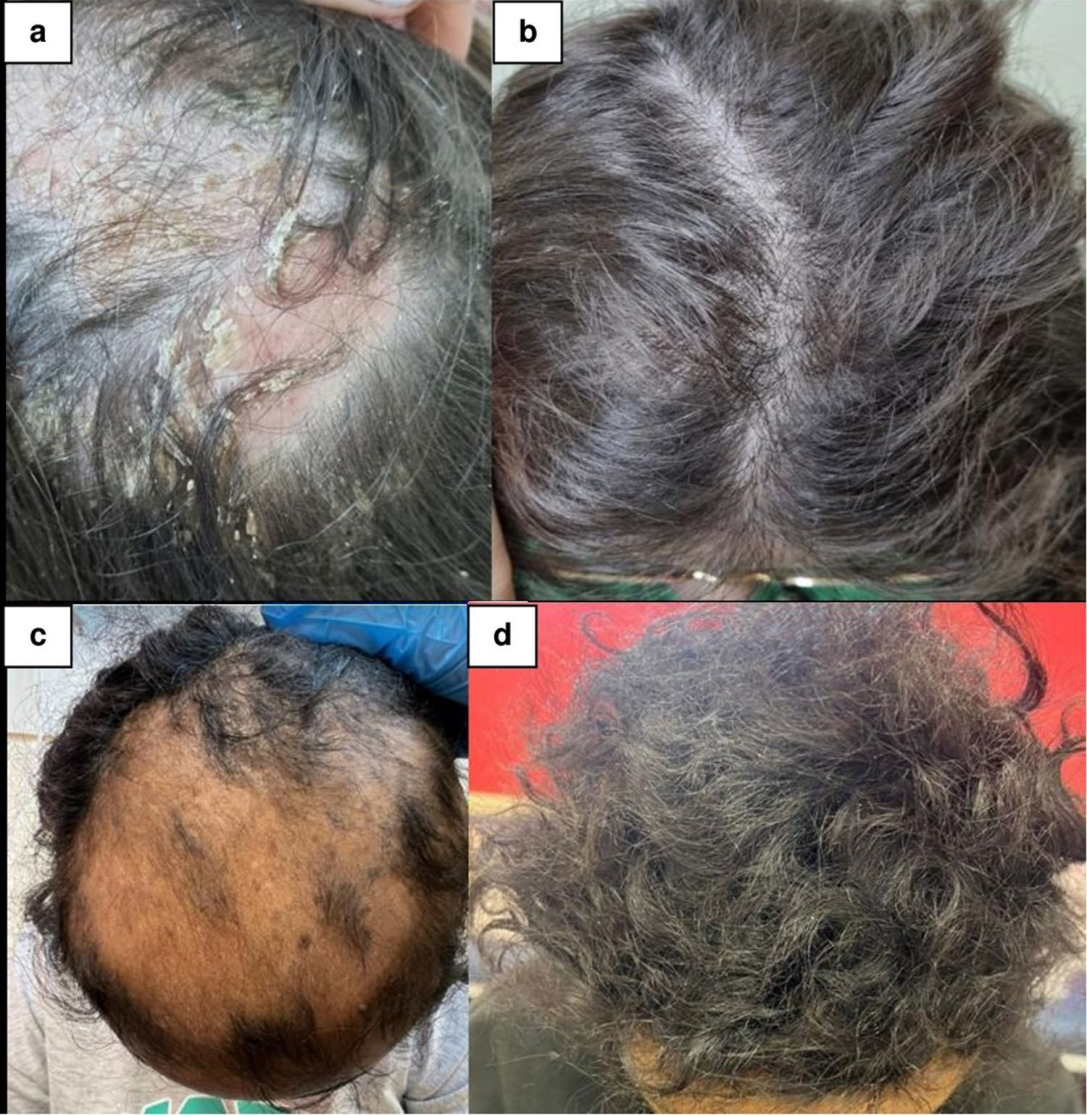
TNFα inhibitor-induced alopecia. a + b patient #8. a- Inflammatory alopecia appearing 13 months after infliximab initiation in a 12yo female diagnosed with Crohn’s disease. b- Recovery after switching treatment to ustekinumab. c + d patient #9. c- Alopecia areata-like eruption appearing 12 months after adalimumab initiation in a 16yo female, diagnosed with Crohn’s disease. d- Regrowth after switching treatment to risankizumab

IA was clinically diagnosed in 18 patients, and AA-like findings were recorded in two patients (Fig. 1; a & b patient #8, 12 year-old female diagnosed with CD. a- inflammatory alopecia appearing 13 months after infliximab initiation, b- recovery after switching treatment to ustekinumab; c & d patient #9, 16 year-old female with CD. c- alopecia areata-like eruption appearing 12 months after adalimumab initiation and d- regrowth after switching treatment to risankizumab).

IA patients also presented with cutaneous psoriasiform eruption (ten patients), follicular psoriasiform eruption (three patients), or both (five patients). Patients who presented with AA-like lesions did not have concomitant cutaneous eruption, but experienced extensive hair loss (> 50% of scalp hair) and an ophiasis-like pattern.

Alopecia treatments included topical steroids (sixteen patients), methotrexate (eight patients), systemic antibiotics (six patients), and acitretin (one patient), all failed to induce hair regrowth within three months or more.

Alternative treatments for baseline GI or rheumatological diseases instead of TNFαI were initiated in 17 patients: ustekinumab in six patients; risankizumab in four patients; tofacitinib in three patients, vedolizumab in two patients, and upadacitinib and secukinumab in one patient each. Of these, 16 patients presented complete resolution, and one patient presented partial hair regrowth—all within six months, without additional therapies for their alopecia.

Additional three patients were recently recommended to switch to another therapeutic class, after TNFαI with concomitant methotrexate and/or acitretin did not result in any improvement. No improvement was observed in patient #14 in Table 1 despite switching from infliximab to adalimumab and adding methotrexate.

Reactivation of baseline inflammatory disease after TNFαI switch was recorded in four patients: three patients with CD treated with ustekinumab and one patient with JIA treated with secukinumab. As a result, one patient increased ustekinumab dosage, one switched to rizankizumab, and one to upadacitinib. Upadacitinib was also administered to the JIA patient instead of secukinumab. These therapeutic changes resulted in baseline disease remission in all patients, along with sustained alopecia resolution. Mean follow-up period was 19.4 ± 10.45 months (range 12–48 months).

Our literature review yielded eight studies describing nineteen pediatric patients [2, 4, 5, 8, 14, 24, 26, 27]. Data regarding demographic and clinical characteristics, management, and outcomes are presented in Table 2.

**Table 2:** Literature review

Reference, year	Sex/age at alopecia onset (years)	Inflammatory condition	TNFα inhibitor	Clinical presentation	Tx duration until alopecia onset (months)	Management	Outcome
Perman et al., 2012 [23]	F/7	JIA	Adalimumab	IA	9	Cys A, MTX, topical TX	PR
	M/11	CD	Infliximab	IA	26	Continued infliximab, topical TX	CR
	M/16	UC	Infliximab	IA	5	Continued infliximab for 10 months, switched to adalimumab, Topical TX	CR
	F/14	CD	Infliximab	IA	2	MTX, topical TX	CR
	F/18	CD	Infliximab	IA	7	Continued infliximab, topical TX	NR
Prata Ribiero et al., 2015 [25]	F/12	CD	Infliximab	IA	4	Continued infliximab, topical TX	CR
Eickstaedt et al., 2017 [8]	F/11	CD	Infliximab	AA	12	Ustekinumab	CR
Campbell et al., 2018 [4]	F/5	CRMO	Infliximab	IA	5	Golimumab	CR
	F/12	CRMO	Infliximab	IA	2	MTX, pamidronate	CR
	F/11	CRMO	Adalimumab	IA	4	Pamidronate	CR
	F/11	CRMO & CD	Infliximab	IA	4	Adalimumab, topical TX	CR
	F/8	CRMO	Infliximab	IA	3	Pamidronate	CR
Groth et al., 2019 [14]	F/14	JIA	Adalimumab	IA	29	Tocilizumab	CR
	F/13	JIA	Infliximab	IA	12	MTX	CR
	F/11	JIA	Adalimumab	IA	7	Ustekinumab	CR
	F/8	JIA	Infliximab	IA	31	Adalimumab, MTX	PR
Sánchez-Pujol et al., 2020 [26]	F/10	Psoriasis	Adalimumab	AA	2	Oral Csx, topical minoxidil	NR
Carrasquillo et al., 2021 [5]	F/12	CD	Adalimumab	IA	6	Ustekinumab	CR
Bonomo et al. 2020 [2]	F/10	CD	Infliximab	IA	12	MTX, Ustekinumab	CR

*AA* alopecia areata, *Abx* antibiotics, *CD* Crohn’s disease, *CR* complete remission, CRMO chronic recurrent multifocal osteomyelitis, *Csx* corticosteroids, *Cys A* cyclosporine A, *F* female, *Foll* follicular, *IA* inflammatory alopecia, *IBD* inflammatory bowel disease, *JIA* juvenile idiopathic arthritis, *M* male, *MTX* methotrexate, *N/A* not available, *NR* no remission, *PR* partial remission, *Tx* treatment, *UC* ulcerative colitis

Male:female ratio was 1:8.5, mean age at alopecia diagnosis was 11.3 ± 3.1 years. Thirteen patients were treated with infliximab and six with adalimumab. Seventeen presented with IA and two with AA-like lesions, after a mean TNFαI treatment duration of 9.6 ± 9.1 months.

Eight patients had IBD: seven CD and one UC. Five patients were diagnosed with JIA, four with chronic recurrent multifocal osteomyelitis (CRMO), psoriasis and concomitant CRMO with CD in one patient each.

Treatment replacement was recommended for 13 patients, of which 11 were reported to exhibit complete hair regrowth, one had partial hair regrowth, and one patient had no response following TNFαI discontinuation.

Four patients continued TNFαI, and three of them had complete hair regrowth. Two patients switched from infliximab to adalimumab, one of them exhibited complete hair regrowth and one had partial response.

## Discussion

TNFαI-induced alopecia is a rare adverse event in both adult and pediatric patients [1, 2, 4, 5, 7, 8, 10, 14, 21, 23, 25, 26, 28, 30]. In this study we describe a relatively large cohort of 20 pediatric patients presenting with psoriasiform IA and AA-like lesions during adalimumab or infliximab treatment.

Psoriatic alopecia is a well described clinical finding in patients with scalp psoriasis. It is attributed to hair loss in tufts when thick psoriatic scales are shed from the scalp [12]. Although TNFαI-induced psoriasiform dermatitis in children often involves the scalp [9], it rarely causes alopecia. Therefore, we presume that in the case of TNFαI- induced alopecia the pathogenesis is not related to a physical factor, but rather to a local inflammatory dysregulation in or around the hair follicle.

In this cohort, two patients presented with AA-like lesions. This adverse event is sporadically reported in children [6, 8, 26], even in comparison with adults [10, 24, 28]. The higher prevalence in adults may be attributed to the fact that more adults are treated with TNFαI, or to the use of concomitant medications for other medical conditions in adult patients, causing further cutaneous inflammatory insult. It is also possible that there is under-diagnosis or under-reporting of this condition in children. Additionally, AA may be more prevalent in adult patients with IBD [22], especially patients treated with TNFαI [22]. Thus, it could be argued that AA cases in this cohort were not drug-induced, but a co-morbidity, potentially triggered by IBD treatment. However, although our patients presented clinical findings associated with negative AA outcomes with extensive hair loss (> 50% of scalp hair) and an ophiasis-like pattern, they experienced an unusual rapid and full response, within weeks, to TNFαI discontinuation. Moreover, their next line of treatment was an IL-23 inhibitor, which is not considered an effective AA therapeutic, reinforcing that AA was induced by TNFα inhibition.

Comparison of our cohort to nineteen pediatric patients previously reported in the literature portrays some similar features [2, 4, 5, 8, 14, 23, 25, 26]. There is a female predominance in both groups (12/20, 60.0% in our cohort and 17/19, 89.5% in previous reports), and most of the patients are adolescents aged ≥ 11 years (18/20, 90.0% in our cohort, 13/19, 68.4% in previous reports). In addition, IBD was the leading diagnosis: 17/20, 85.0% in our cohort and 8/19, vs 42.1% in previous reports.

The female predominance might be attributed to the fact that immune-mediated diseases typically show a female preponderance. Alternatively, girls may potentially be more concerned by the appearance of alopecia and seek treatments more than male patients.

In both our cohort and in the literature, AA-like lesions were similarly rare (2/20, 10.0% and 2/19, 10.5%, respectively), but reported in larger numbers in adults [1, 10, 28]. The reason for these specific demographic characteristics is not clear and could be attributed to the fact that some adverse events tend to effect certain populations, as is the case for other medications. For example, acneiform eruption appears in adolescents but rarely in pre-adolescent children treated with MEK inhibitors [3, 11].

Duration of TNFαI treatment before alopecia onset differed between our cohort and previous reports. While 75.0% of our patients presented with alopecia at least 10 months after treatment initiation, 68.4% of patients reported in the literature presented with alopecia during the first nine months of TNFαI treatment. Considering the wide variability in the timing of alopecia onset after TNFαI therapy initiation, alopecia should be suspected as a potential drug-induced adverse event in these patients, regardless of whether it occurs early or later during TNFαI treatment.

In our cohort, 50% of patients were treated with adalimumab and 50% with infliximab, in contrasts with previous reports, where infliximab was the predominant medication associated with alopecia (16 out of 19 cases). This discrepancy may reflect the frequent use of infliximab in the past, whereas both infliximab and adalimumab are now widely used. The equal distribution of these treatments in our cohort supports the rational that alopecia associated with TNFαI is likely a class effect, attributable to the shared mechanism of action of these agents.

In previous reports, treatment replacement resulted in hair regrowth in all patients but one. Our findings are in line with these reports, with 17/20 (85.0%) of our patients switching to other treatments, resulting in complete resolution in 16 of them and partial hair regrowth in one. It is noteworthy that even patients who received vedolizumab for their IBD as a substitute for TNFαI showed complete alopecia resolution, although vedolizumab has no effect on alopecia areata. Taken together, these data provide strong rationale for treatment replacement as a key for recovery. On the other hand, three out of four patients in the literature who continued TNFαI had complete hair regrowth, while in our cohort patients remaining on TNFαI therapy had recalcitrant alopecia. Moreover, limited previous studies reported that using an in-class substitute medication, i.e., switching from infliximab to adalimumab, can also result in hair regrowth.

While TNFαI cessation was associated with the best clinical outcomes for alopecia, it is still questionable whether switching baseline treatment is always recommended in these patients. In our cohort, 36% of patients with a well-controlled inflammatory gastrointestinal or rheumatological disease experienced disease worsening upon TNFαI replacement, necessitating additional therapeutic adjustments. Switching an effective treatment is a decision that should be taken in solemnity, and preferably by a multi- disciplinary team. Nevertheless, nowadays newly developed targeted medications with high efficacy are available, making this decision somewhat easier.

It is noteworthy that 90% of the patients in our cohort also presented with cutaneous eruption, 44% of them with follicular psoriasiform eruption. Follicular psoriasiform dermatitis induced by TNFαI was reported in a few cases [13]. It is possible that this unique presentation is under-diagnosed, and not necessarily related to the alopecia.

The pathophysiology of TNFα inhibitor-induced alopecia in not fully understood. Presumably, the inhibition of TNFα causes increased interferon α expression and homing of Th1 cells to the skin, resulting in psoriasiform dermatitis [7, 23]. In addition, TNF-α blockade induces a Th17 immune response and down-regulation of Treg cells, which are dysfunctional in alopecia areata [29].

The limitations of this study are its retrospective design, selection bias, lack of data and lab results regarding disease activity during the alopecia appearance and after the recovery, lack of standardized outcome measures for this entity and the small sample size.

In the era of targeted medicine, new medications offer expanded treatment options for patients but are also associated with emerging cutaneous side effects. This emphasizes the need for a standardized, validated grading system for drug-induced alopecia. Such a system would enable more accurate assessment of severity and support the development of evidence-based treatment algorithms.

In conclusion, this study includes, to our best knowledge, the largest single center cohort of pediatric patients with TNFαI-induced alopecia. Although switching from TNFαI therapy appeared to be key for hair regrowth, it was often associated with reactivation of baseline inflammatory diseases. Our findings highlight the need for further research to identify and establish the most effective approach for this challenging adverse event.

## Data Availability

The data underlying this article cannot be shared publicly due to the privacy of individuals that participated in the study. Data will be provided upon reasonable request.
